# Rasch analysis of the Chinese version of the clinically useful depression outcome scale in patients with major depressive disorder

**DOI:** 10.1186/s40359-023-01255-7

**Published:** 2023-08-02

**Authors:** Jing Zhong, Hai-Yan Ma, Xue-Mei Wang, Xiao-Jie Huang, Ming-Zhi Xu

**Affiliations:** 1 School of Public Health, Southern Medical University, Guangzhou, Guangdong People’s Republic of China; 2 Guangdong Mental Health Center, Guangdong Provincial People’s Hospital (Guangdong Academy of Medical Sciences), Southern Medical University, Guangzhou, 510120 Guangdong People’s Republic of China; 3 Guangdong second provincial general hospital, Guangzhou, Guangdong People’s Republic of China; 4 The Second Clinical College, Guangdong Provincial Hospital of Traditional Chinese Medicine, Guangdong Provincial Academy of Chinese Medical Sciences, Guangzhou University of Chinese Medicine, Guangzhou, People’s Republic of China

**Keywords:** Depression, Clinically useful Depression Outcome Scale (CUDOS), Psychometric properties, Rasch analysis

## Abstract

**Objective:**

To examine the psychometric properties of the Chinese version of the Clinically Useful Depression Outcome Scale (CUDOS) in the Chinese patients with major depressive disorder (MDD) using Rasch analysis.

**Methods:**

The sample consisted of 283 patients with MDD (69% females). The Rasch model was applied to examine the overall fit of the Chinese version of CUDOS and the fit of the 18 items. Dimensionality, item-model fit, differential item functioning (DIF), reliability, ordering of response category and targeting were tested to examine the psychometric properties of the Chinese version of CUDOS.

**Results:**

Rasch analysis demonstrated the unidimensionality of the Chinese version of CUDOS. Of the 18 items, three items (item 4, item 5, item 6) showed misfit in the model. After merging item 4 into item 3 and item 6 into item 5, the overall model fit improved. The person separation index (PSI) was 3.0 and the person reliability coefficient was 0.90. No evidence of significant DIF was found when associated with gender and age. No disordered category and threshold of the rating response were observed, which meant the response category setting was reasonable. The mean ability of person was − 0.53.

**Conclusion:**

The results suggested that the Chinese version of CUDOS has acceptable psychometric properties. In order to improve the quality and applicability of the Chinese version of CUDOS, the merging of item 4 into item 3 and item 6 into item 5 are suggested.

## Introduction

Major depressive disorder (MDD) is a common mental disorder with high rates of morbidity, recurrence, disability and suicide, which has become a significant public health problem of worldwide concern [1–4]. Approximately 322 million people suffer from depression around the world, which is 4.4% of the global population [5]. A cross-sectional epidemiological study showed that the lifetime prevalence of MDD in mainland China was about 3.4% [6]. Moreover, World Health Organization (WHO) predicted that MDD would become a leading cause of the global burden of disease by 2030 [7]. Remission is recognized as the optimal outcome of treatment for depression. However, low rates of remission and high rates of relapse commonly appear in clinical consequences, which contribute to impaired social function and reduced quality of life [8, 9]. In clinical practice, measurement-based care (MBC) has proved to be of great benefit in the treatment for patients with MDD. Clinicians can be able to adjust treatment strategies by referring to the results of measurements, which may promote treatment outcomes [10]. Therefore, a reliable and valid instrument to measure clinical outcomes for patients with MDD is required.

Clinically Useful Depression Outcome Scale (CUDOS) is a brief self-administered depression questionnaire developed by Zimmerman that contains 18 items. It is easy to use that takes around less than 3 min to complete and less than 15 s to score. The CUDOS was designed according to the Diagnostic and Statistical Manual of Mental Disorders, Fourth Edition (DSM-IV) criteria for MDD, proved to be clinically useful and sensitive to the changes of depressive symptoms [11]. In addition, the CUDOS evaluates not only severity of depressive symptoms but also psychological impairment and life quality, which provides clinicians with more useful information of treatment outcomes [12]. The CUDOS is also user-friendly, it has less burden of scale completion to patients so they are willing to complete it regularly [13]. The original English version of CUDOS has been translated into multiple languages and all these translated versions have consistently demonstrated good reliability and validity [14–17].

In our previous study, we examined the psychometric properties of the Chinese version of CUDOS in patients with MDD using traditional Classical Test Theory (CTT). Depressive symptoms were assessed in 190 patients with MDD using the Chinese version of the CUDOS, 17-item Hamilton Depression Rating Scale (HRSD) and the modified Overall Clinical Impression-Severity Scale 15 (iCGI-S). Reliability, validity tests, and receiver operating characteristic curves were performed. The result showed that the Chinese version of CUDOS was of great value as a brief and reliable tool to assess depressive symptoms and clinical outcome [14]. However, the result reflected only the overall performance of the Chinese version of CUDOS, because of the limitations of CTT, it could not provide detailed information of individual item performance. Moreover, the test dependence and sample dependence of CTT, may also contributed to non-objectivity of the measurement results [18, 19]. The Item Response Theory (IRT) was developed to compensate for the limitations of CTT. Rasch model is one of the IRT models that emphasizes one-parameter and unidimensional research paradigm, which is relatively simple comparing to other IRT models [20–22]. In recent years, the application of Rasch analysis in health outcome measures has become popular [23–25]. It has advantages comparing to CTT, including displaying test and sample independence, providing linear transformation of the ordinal raw score and diagnostic details on how the scale can be improved by exploring the performance of individual items [23, 26].

The purpose of the current study was to evaluate the psychometric properties of the Chinese version of CUDOS by using Rasch analysis. Dimensionality, item-model fit, differential item functioning (DIF), reliability, response category ordering, and targeting were assessed in patients with MDD to diagnose potential measurement problems and to make recommendations for improving the quality and applicability of the Chinese version of the CUDOS in patients with MDD.

## Methods

### Participants

The participants consisted of 283 patients with MDD recruited from Guangdong Mental Health Center in China between October 2018 and August 2021. Patients were included if the following criteria were met: (1) diagnosis of MDD based on the Diagnostic and Statistical Manual of Mental Disorders, Fifth Edition (DSM-5), and it was diagnosed by two psychiatrists with attending or above professional titles;(2) the aged from 18 to 65 years old;(3) all patients signed an informed consent form. Patients were excluded if the following criteria were met: (1) patients suffering from other mental disorders (such as bipolar disorder, etc.);(2) patients suffering from severe physical illness;(3) patients with a history of substance abuse (e.g.alcohol and drugs) within the past year;(4) women in pregnancy or breastfeeding.

### Procedures

First, a psychiatrist with an attending title or higher confirmed the participants’ diagnosis according to the DSM-5. Followed by an interview with MDD patients by a trained researcher, who presented the intent and content of the study to patients with MDD. Patients with MDD voluntarily participated and signed an informed consent form. Patients with MDD who met the inclusion and exclusion criteria were included in this study. Subsequently, general demographic and clinical characteristics (e.g., gender, age, marital status, family history, duration of depression and years of education, etc.) were collected from patients with MDD. Finally, patients with MDD completed a self-report scale assessment in a quiet room.

### Instrument

The CUDOS was a self-report questionnaire for assessing the depressive symptoms and identifying remission status according to the DSM-IV [13, 27]. The CUDOS consisted of 18 items evaluating all of the DSM-IV criteria for MDD as well as psychosocial impairment and the impact of depressive symptoms on life quality. Each item was assessed by utilizing a 5-point Likert-type scale. Patients chose a number according to their condition during the past week (including today): 0 = not at all true/0 days; 1 = rarely true/1–2 days; 2 = sometimes true/3–4 days; 3 = usually true/5–6 days; 4 = almost always true/everyday, with total scores ranging from 0 to 72 points [11]. In the current study, the Chinese version of the CUDOS was used to assess depressive symptoms. Good reliability and validity of the scale have been demonstrated through CTT method [14].

### Statistical analysis

The collected data were analyzed by descriptive statistics using SPSS version 26.0 (IBM Corporation, Chicago, IL). WINSTEPS 4.8.2 software was used to conduct Rasch analysis. The analysis included dimensionality, item-model fit, DIF, reliability, ordering of response categories and targeting. The sample size was based on definitive or high stakes with best to poor targeting sample size of exceeding 250 samples at 99% confidence [28].

#### Unidimensionality

In Rasch analysis, dimensionality was analyzed by principal component analysis (PCA). The eigenvalue of the first contrast was suggested to be between 1.4 and 2.1 [29, 30]. In addition,a proportion of variance that could be explained by Rasch model exceeded 50% indicated the construct of the scale was unidimensional [31]. Pearson correlations > 0.57 was suggested to be acceptable [32]. While Rasch model divided the items into several clusters, the high disattenuated correlation (r > 0.3) between 2 clusters indicated that these clusters might perform a same dimension.

#### Item-model fit

Wright and Linacre(1994) suggested the values of information-weighted mean-square fit statistic (infit MnSq) and outlier-sensitive mean-square fit statistics (outfit MnSq) were closer to 1, indicating that data fitted Rasch model [18]. For clinical purposes, the values of infit and outfit MnSq of individual item should be between 0.5 and 2.0, or else it would be considered as a misfitting item [18]. Item-model fit could also be evaluated graphically by the Item Characteristic Curve (ICC), which indicated to fit well if plots fall on the expected curve [33].

#### Differential item functioning (DIF)

DIF analysis was conducted to identify the systematic and random bias of the measurement [34]. DIF was assessed by comparing participants in different groups matched for trait levels. In this study, patients with MDD were categorized based on gender (female/male) and age (split at median:18–26 y/27–65 y), respectively. The DIF contrast > 0.64 was considered to be notable [35].

#### Reliability

Item reliability and person reliability indices should fall between 0 and 1 [33]. The item separation index (ISI) and person separation index (PSI) must exceed 2.0 to ensure the separation reliability coefficient to be above 0.8. In addition, person reliability coefficient was associated with Cronbach’s ɑ [36]. The Cronbach’s ɑ exceeding 0.8 was considered to be satisfactory [36].

#### Ordering of response category

To test the ordering of response categories we examined fit values and average measures of the categories and thresholds. The category probability curves (CPC) provided visualization of response category function [33]. The infit MnSq and outfit MnSq statistics should be between 0.5 and 1.7. Moreover, average category measures should monotonically increase with categories [33]. The thresholds between adjacent categories should be between 1.4 and 5 logits, and monotonically increased with categories [33].

#### Targeting

Item-person map (Wright map) displayed the item location and person location on the same logit scale [33]. Differences of greater than 1.0 logits between person mean measures and item mean measures were considered to be notably mistargeting [33, 37]. Items with logit values below 0 were relatively easier, and items with logit values exceed 0 were relatively difficult [33].

## Results

### Participant characteristics

A total of 298 patients with MDD were investigated. 15 patients were excluded for lacking of sociodemographic and clinical characteristics. Finally, 283 patients were enrolled in this study, of which 31.1% were male and 68.9% were female. The age range from 18 to 61 with a mean age of 29.02 years and a standard deviation (SD) of 0.60 years. Besides, 68 (24.0%) patients had family history of mental disorders, and 171 (60.4%) were first-episode patients. Table 1 showed the demographic information and the clinical characteristics.

**Table 1:** Demographic and Clinical Characteristics

Varieble	Total (N = 283)
Sex, n (%)	
Male	88 (31.1%)
Female	195 (68.9%)
Marry status, n (%)	
married	102 (36.0%)
unmarried	181 (64.0%)
Race, n (%)	
Han	277 (97.9%)
No-Han	6 (2.1%)
First episode, n (%)	
Yes	171 (60.4%)
No	112 (39.6%)
Family history, n (%)	
Yes	68 (24.0%)
No	215 (76.0%)
Antidepressant treatment, n (%)	
Yes	179 (63.3%)
No	104 (36.7%)
Average age (years), M (SD)	29.02 (10.02)
Range	18–61
Education (years), M (SD)	13.56 (3.26)
Onset age (years), M (SD)	25.64 (9.31)
Duration of illness for MDD(years), M (SD)	3.38 (4.48)
CUDOS, M (SD)	29.64(17.56)

Note: M, mean; SD, standard deviation

#### Unidimensionality

**Fig. 1:**
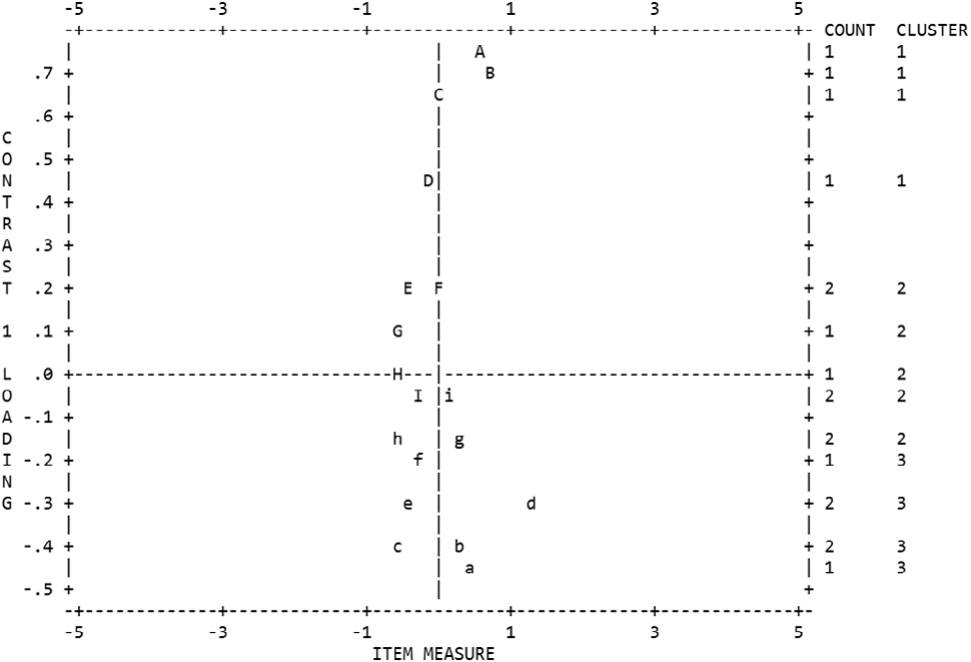
Screen plot of loadings for the first contrast

The eigenvalue of the first contrast was 2.6. The proportion of raw variance data that can be explained by Rasch model was 56.7%. In addition, the rasch analysis divided the data into 3 clusters (Fig. 1). The person correlations between 3 clusters of items ranged from 0.62 to 0.79, and the disattenuated correlations between 3 clusters of items ranged from 0.89 to 1.00 (Table 2).

**Table 2:** Person and item summary statistics

	Item (N = 18)	Person (N = 283)
Mean measure	0.00	−0.53
Infit MnSq	1.03	1.01
Infit ZSTD	−0.10	−0.70
Outfit MnSq	1.11	1.11
Outfit ZSTD	−0.50	0.00
Separation index	7.03	3.00
Reliability index	0.98	0.90
Clusters	Person correlations	Disattenuated correlations
1–2	0.62	0.89
1–3	0.79	1.00
2–3	0.75	0.96

Note: Infit MnSq, information-weighted mean-square fit statistic; Outfit MnSq, outlier-sensitive mean-square fit statistics; ZSTD, standardized mean square residual fit statistic

Items above the zero horizontal line indicate positive loading and items below indicate negative loading.

#### Item-model fit

**Fig. 2:**
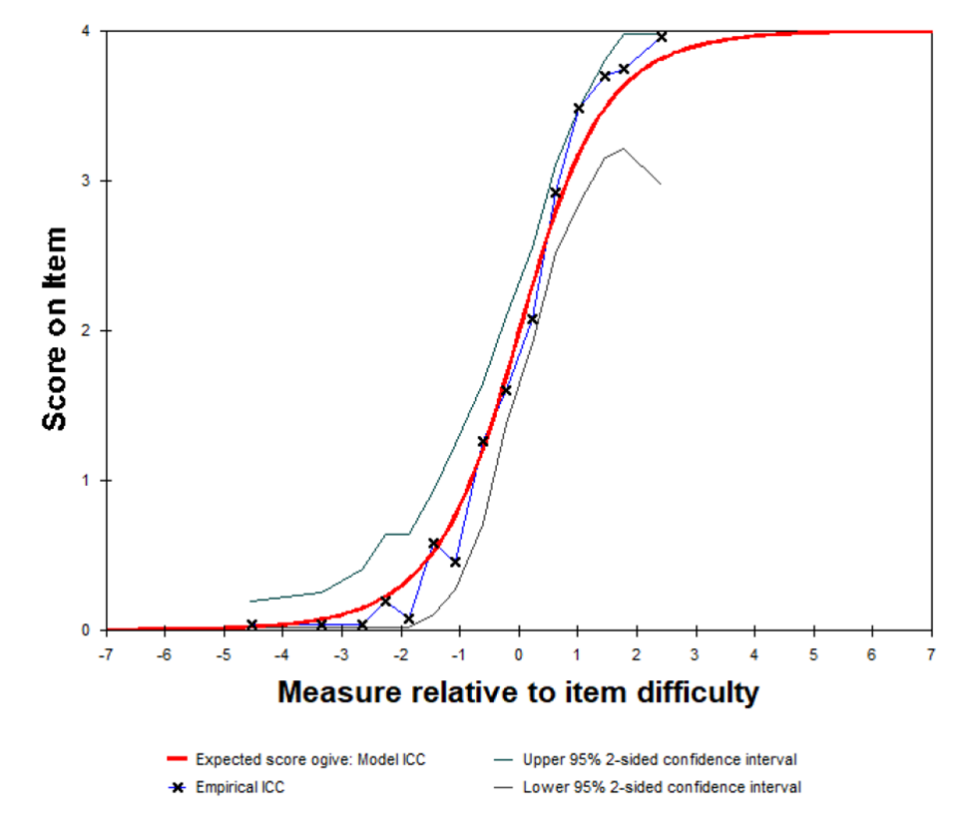
Item characteristic curve

Overall infit and outfit statistics of items and person were close to 1 (Table 2). The great majority of individual items had infit and outfit MnSq between 0.5 and 1.7. But the outfit statistics of item 4 (My appetite was much greater than usual), item 5 (I had difficulty sleeping) and item 6 (I was sleeping too much) were outside this range. The outfit statistics values of item 4, item 5 and item 6 were 3.82, 1.85 and 2.57, respectively, and the infit statistics values of item 4, item 5 and item 6 were 2.09, 1.75 and 2.30, respectively (Table 3). The overall fit statistic was presented in Table 2 and the individual items fit statistic was presented in Table 3. The ICC showed that most of plots fell on the expected curve and all of these points were within the 95% confidence interval (Fig. 2). After merging item 4 into item 3 and item 6 into item 5, the overall model fit improved (Table 4) and the infit and outfit statistics for all individual items of the scale were with in the acceptable range (Table 5).

**Table 3:** Summary table of item-model fit statistics, item measure differential item functioning statistics

Item description	PCA loading	Measure (logit)	Item fit				DIF contrast	
			Infit MnSq	Infit ZSTD	Outfit MnSq	Outfit ZSTD	Gender	Age
CUDOS								
1.I felt sad or depressed	0.20	-0.40	0.53	-6.91	0.50	-5.99	-0.19	0.11
2.I was not as interested in my usual activities	0.09	-0.53	0.65	-4.84	0.63	-4.20	-0.26	0.00
3. My appetite was poor and I didn’t feel like eating	-0.07	0.14	1.21	2.45	1.29	2.39	0.22	0.07
4. My appetite was much greater than usual	-0.32	1.28	2.09	7.89	3.82	9.59	0.10	-0.09
5. I had difficulty sleeping	-0.22	-0.26	1.75	7.59	1.85	6.60	-0.13	0.47
6. I was sleeping too much	-0.47	0.45	2.30	9.90	2.57	8.85	0.30	-0.32
7. I felt very fidgety, making it difficult to sit still	-0.16	0.34	0.83	-2.09	0.79	-1.84	-0.15	0.25
8. I felt physically slowed down, like my body was stuck in mud	-0.39	0.23	0.94	-0.65	0.82	-1.60	0.04	0.09
9. My energy level was low	-0.39	-0.53	0.70	-4.05	0.64	-4.00	-0.11	0.00
10. I felt guilty	0.20	-0.01	0.79	-2.76	0.77	-2.31	0.03	-0.16
11. I thought I was a failure	0.45	-0.16	0.87	-1.71	0.77	-2.29	-0.02	-0.05
12. I had problems concentrating	-0.28	-0.44	0.72	-3.69	0.68	-3.49	0.10	0.00
13. I had more difficulties making decisions than usual	-0.07	-0.22	0.76	-3.26	0.74	-2.71	0.04	-0.06
14. I wished I was dead	0.75	0.56	1.15	1.69	0.92	-0.58	0.55	-0.30
15. I thought about killing myself	0.71	0.71	1.13	0.95	0.60	0.58	0.42	-0.20
16. I thought that the future looked hopeless	0.67	-0.05	0.89	0.76	0.74	0.68	0.04	-0.31
17.Overall, how much have symptoms of depression interfered with or caused difficulties in your life during the past week?	0.02	-0.52	0.64	0.65	0.80	0.73	-0.04	0.11
18.How would you rate your overall quality of life during the past week?	-0.14	-0.59	0.52	0.84	0.79	0.74	-0.17	0.22

Note: Infit MnSq, information-weighted mean-square fit statistic; Outfit MnSq, outlier-sensitive mean-square fit statistics; ZSTD, standardized mean square residual fit statistic; PCA, principal component analysis; DIF, differential item functioning; CUDOS, Clinically Useful Depression Outcome Scale

**Table 4:** Person and item summary statistics after merge item 4 into item3 and merge item 6 into item 5

	Item (N = 16)	Person (N = 283)
Mean measure	0.00	−0.57
Infit MnSq	1.02	1.00
Infit ZSTD	0.00	−0.10
Outfit MnSq	1.00	1.00
Outfit ZSTD	−0.30	−0.10
Separation index	7.26	3.26
Reliability index	0.98	0.91

Note: Infit MnSq, information-weighted mean-square fit statistic; Outfit MnSq, outlier-sensitive mean-square fit statistics; ZSTD, standardized mean square residual fit statistic

**Table 5:** Item-model fit statistic after merging item 4 into item 3 and item 6 into item 5

Item description	Item fit			
	Infit MnSq	Infit ZSTD	Outfit MnSq	Outfit ZSTD
CUDOS				
1.I felt sad or depressed	0.65	-4.77	0.60	-4.66
2.I was not as interested in my usual activities	0.79	-2.76	0.75	-2.76
3. My appetite was poor and I didn’t feel like eating or my appetite was much greater than usual	0.98	-0.56	1.37	2.49
4. I had difficulty sleeping or I was sleeping too much	1.48	5.84	1.81	5.84
5. I felt very fidgety, making it difficult to sit still	1.08	0.15	1.01	0.15
6. I felt physically slowed down, like my body was stuck in mud	1.21	2.35	0.82	0.39
7. My energy level was low	0.89	-1.38	1.04	-1.99
8. I felt guilty	1.01	0.12	0.96	-0.30
9. I thought I was a failure	1.07	0.82	0.95	-0.43
10. I had problems concentrating	0.92	-1.00	0.86	-1.50
11. I had more difficulties making decisions than usual	0.95	-0.64	0.93	-0.70
12. I wished I was dead	1.41	4.08	1.12	-0.86
13. I thought about killing myself	1.38	3.75	1.16	1.08
14. I thought that the future looked hopeless	1.08	0.94	0.92	-0.69
15. Overall, how much have symptoms of depression interfered with or caused difficulties in your life during the past week?	0.79	-2.63	0.76	-2.59
16.How would you rate your overall quality of life during the past week?	0.66	-4.58	0.98	-0.15

Note: Infit MnSq, information-weighted mean-square fit statistic; Outfit MnSq, outlier-sensitive mean-square fit statistics; PCA, principal component analysis; DIF, differential item functioning; CUDOS, Clinically Useful Depression Outcome Scale

The most of plots fell on the expected curve and all of these plots were within the 95% confidence interval.

#### Differential item functioning (DIF)

DIF analysis was performed to detect whether DIF existed according to gender (female/male) and age (18–26 y/27–65 y). Results showed that there was no DIF contrast statistics greater than 0.64 (Table 3).

#### Reliability

The ISI and the PSI statistics were 7.03 and 3.00 respectively (Table 2). Besides, the item reliability coefficient was 0.98 and the person reliability coefficient was 0.90 (Table 2). The reliability of all items were at an acceptable level.

#### Ordering of response category

**Fig. 3:**
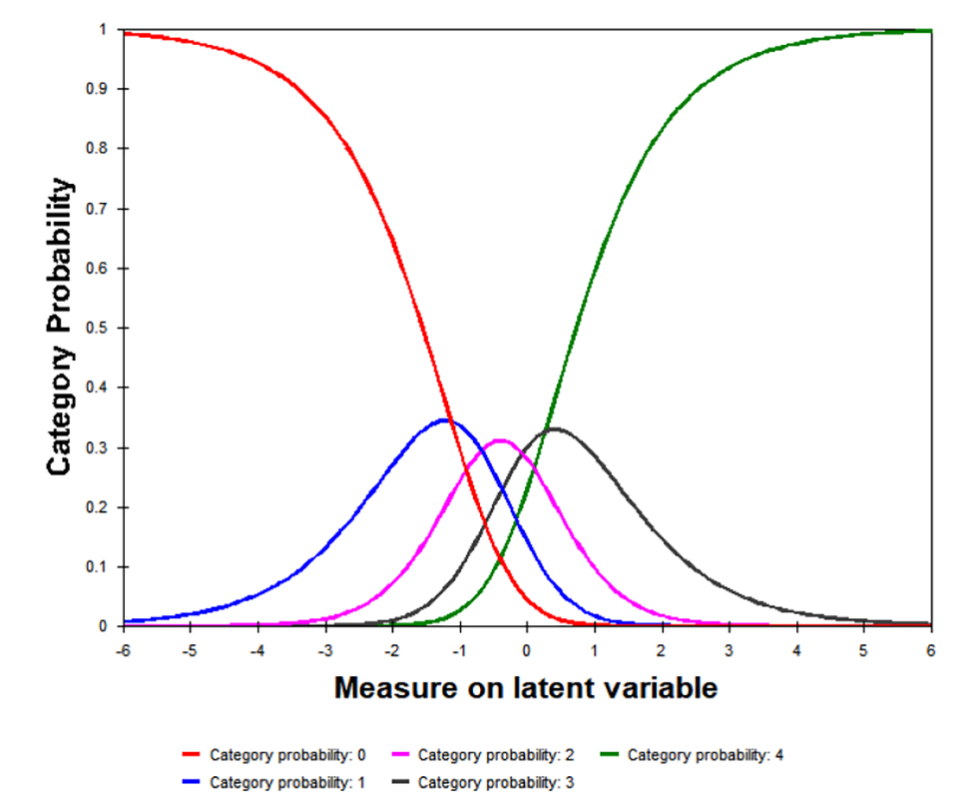
Category probability curve showing ordered thresholds

The summary of category structure statistics presented in Table 6. All category infit MnSq and outfit MnSq statistics were in the range of 0.5 to 1.7. And the average measure increased monotonically from − 2.17 to 2.14. The threshold increased monotonically as well with category from − 0.73 to 0.67. However, the thresholds differences between adjacent categories were less than 1.4. The CPC showed that all thresholds were ordered (Fig. 3).

**Table 6:** Summary of category structure statistics

Cat. Label	Infit MnSq	Outfit MnSq	Threshold	Average Measures
0	1.07	1.09	NONE	(−2,17)
1	0.86	0.91	−0.73	−0.81
2	0.89	1.16	−0.28	0.01
3	0.91	1.06	0.33	0.82
4	1.11	1.33	0.67	(2.14)

Note: Cat, category; Infit MnSq, information-weighted mean-square fit statistic; Outfit MnSq, outlier-sensitive mean-square fit statistics; DIF, Differential Item Functioning

Each curve represented the probability of endorsing a response option. The red, blue, pink,black and green curves on the graph represent the 0, 1, 2, 3 and 4 rating categories respectively.

#### Targeting

**Fig. 4:**
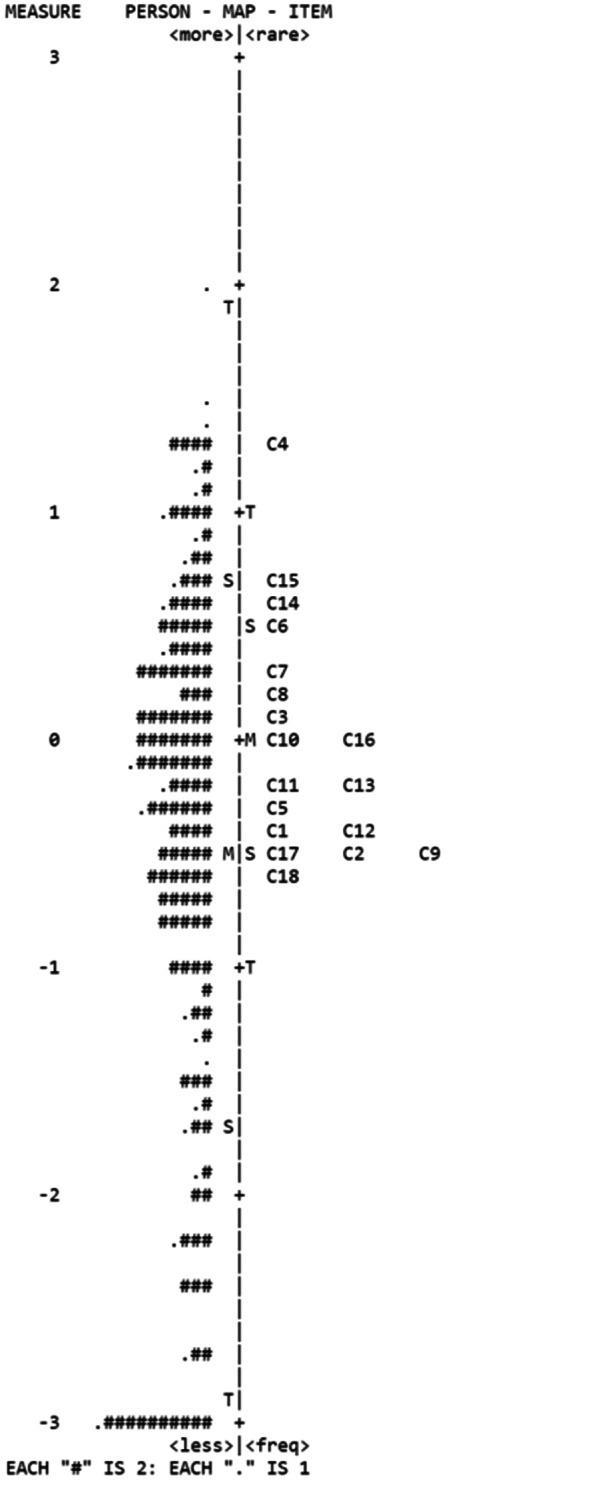
The item-person map of the Chinese version of CUDOS. Participants were located on the left of the dashed line and items were on the right of the dashed line. Each ‘**#**’ and ‘.’ represented three and one participant, respectively. M, mean; S, 1 SD from the mean; T, 2 SD from the mean

All of the individual item measures were between − 0.59 and 1.28 logits, with item 4 (My appetite was much greater than usual) was the hardest item and item 18 (How would you rate your overall quality of life during the past week?) was the easiest item (Table 3). Furthermore, the person mean measure was − 0.53 logits, and the item mean measure was 0 (Table 2). Besides, the item-person map (Wright map) compared the correspondence between the mean person locations and the mean item locations (Fig. 4), and most of items and person locations were near 0.

## Discussion

The Chinese version of CUDOS had been validated in Chinese patients with MDD by CTT methods, which display good content validity, calibration validity, and discriminant validity [14]. Yet there was necessary to confirm the psychometric properties of the Chinese version of CUDOS in detail before it could be widely adopted. Rasch model derived from IRT methods that could provide more details of the measure and compensate the defects of CTT methods [23]. In this study, the Rasch analysis had identified some strengths and limitations of the Chinese version of CUDOS that were not previously observed when using CTT methods.

Firstly, the dimensionality of the Chinese version of CUDOS was tested. The eigenvalue of the first contrast was slightly higher than the criterion, which might indicate a multi-dimensionality. However, the proportion of raw variance data, high disattenuated correlations and person correlations suggested that the Chinese version of CUDOS was a unidimensional contruct scale, which was consistent with those previous studies by CTT methods [11, 14].

As to the fit analysis, the Chinese version of CUDOS demonstrated that the majority of items adequately fitted the model. Three items showed poor outfit statistics (misfitting), the item 4 (My appetite was much greater than usual), item 5 (I had difficulty sleeping) and item 6 (I was sleeping too much).Misfitting items were suggested to be deleted or modified theoretically. Given that difficulty sleeping was a common and important symptom of MDD which should be retained, it was necessary to modify or adjust the language description of item 5. Though item 4 and item 6 were not typical symptoms of MDD, deleting them directly might result in a lack of information for assessment in clinical practice. Since item 3 and item 4 fell in the same category, so did item 5 and item 6. Merging item 4 into item 3 and item 6 into item 5 were considered. The results showed that the overall model fit improved.

To investigate the possibility of item bias, DIF analysis was conducted to determine if items exhibited gender- and age-based DIF. The items did not show evidence of DIF across gender or age in the sample of patients with MDD, indicating that prominent item bias was not found in the Chinese version of CUDOS.

PSI in Rasch analysis associates Cronbach’s ɑ [36]. The original English version of CUDOS demonstrated good reliability with a Cronbach’s ɑ of 0.90 [11]. The obtained results indicated that the Chinese version of CUDOS has good reliability, indicating that the scale was a reliable tool for patients with MDD.

As a proper rating scale, each of the items, respondents with high levels of the attribute being measured are supposed to endorse high scoring responses. The results indicated that the ordering of response categories were reasonable in most aspects, and disordered response categories or thresholds was not found. However, thresholds between adjacent categories were too close to meet the criteria (< 1.4 logits), which indicated that the adjacent response categories were not distinguishing enough. Probably it was because the sample was homogeneous,meanwhile, splitting a week time into five response categories might lead to a narrow margin between adjacent options.

The Rasch analysis transforms raw scores to be interval and then compare person ability and item difficulty in a same logit scale [38, 39]. In clinical practice, the measurement used are appropriately targeted at the population being assessed [40]. The Chinese version of CUDOS total scale person-item map showed that items were evenly distributed around 0. The difference between person mean and item mean measure were less than 1.0 logits. The results indicated the severity of depressive symptoms in patients with MDD could be accurately captured by the items in the Chinese version of CUDOS.

This study had several limitations. Firstly, all participants were recruited at the Guangdong Mental Health Center in China. Further multi-center studies should be conducted in the future to explore the applicability of the Chinese version of CUDOS in patients with MDD. Secondly, the results might be influenced by selection bias, as few middle-aged and elderly subjects were included. Therefore, the data might not represent an accurate cross-sectional study. Moreover, structured interviews were not adopted in this study, which somewhat weakened the strength of homogeneity of the sample. The applicability of the Chinese version of CUDOS in healthy population needs to be further explored. Finally, only 1-parameter logistic model was considered in this study, and 2-parameter logistic model and 3-parameter logistic model can be further used for comparison in the future and try to find the difference of the results.

## Conclusion

In summary, the Chinese version of CUDOS is a reliable tool to evaluate depression symptoms for patients with MDD. Rasch analysis of the Chinese version of CUDOS largely confirmed the unidimensionality of the instrument. There was no notable differential item functioning (DIF) across either gender or age. No disordered response category was found. And the scale had a well-targeted measure. In order to improve the quality and applicability of the scale, it is suggested that item 4 be merged into item 3 and item 6 into item 5.

## Data Availability

Due to ethical restrictions, the present study data were not publicly available to ensure that research participants privacy is not compromised. Data for this study are available from the corresponding author, Dr. Xu.
